# SEGS-1 a cassava genomic sequence increases the severity of African cassava mosaic virus infection in *Arabidopsis* thaliana

**DOI:** 10.3389/fpls.2023.1250105

**Published:** 2023-10-17

**Authors:** Cyprian A. Rajabu, Mary M. Dallas, Evangelista Chiunga, Leandro De León, Elijah M. Ateka, Fred Tairo, Joseph Ndunguru, Jose T. Ascencio-Ibanez, Linda Hanley-Bowdoin

**Affiliations:** ^1^ Department of Plant and Microbial Biology, North Carolina State University, Raleigh, NC, United States; ^2^ Department of Horticulture, Jomo Kenyatta University of Agriculture and Technology, Nairobi, Kenya; ^3^ Department of Molecular and Structural Biochemistry, North Carolina State University, Raleigh, NC, United States; ^4^ Tanzania Agricultural Research Institute-Mikocheni, Dar Es Salaam, Tanzania

**Keywords:** SEGS-1, begomovirus, ACMV, Arabidopsis thaliana, cassava

## Abstract

Cassava is a major crop in Sub-Saharan Africa, where it is grown primarily by smallholder farmers. Cassava production is constrained by Cassava mosaic disease (CMD), which is caused by a complex of cassava mosaic begomoviruses (CMBs). A previous study showed that SEGS-1 (sequences enhancing geminivirus symptoms), which occurs in the cassava genome and as episomes during viral infection, enhances CMD symptoms and breaks resistance in cassava. We report here that SEGS-1 also increases viral disease severity in *Arabidopsis thaliana* plants that are co-inoculated with African cassava mosaic virus (ACMV) and SEGS-1 sequences. Viral disease was also enhanced in Arabidopsis plants carrying a SEGS-1 transgene when inoculated with ACMV alone. Unlike cassava, no SEGS-1 episomal DNA was detected in the transgenic Arabidopsis plants during ACMV infection. Studies using *Nicotiana tabacum* suspension cells showed that co-transfection of SEGS-1 sequences with an ACMV replicon increases viral DNA accumulation in the absence of viral movement. Together, these results demonstrated that SEGS-1 can function in a heterologous host to increase disease severity. Moreover, SEGS-1 is active in a host genomic context, indicating that SEGS-1 episomes are not required for disease enhancement.

## Introduction

Cassava (*Manihot esculenta* Crantz) is a major crop across Africa, providing food and income to over 300 million people. Cassava can be grown on marginal lands and with limited water, but its production is severely limited by viral diseases (OkogBenin et al., 2013). Cassava mosaic disease (CMD) is one of the most important viral diseases of cassava, with yield losses ranging from 20 to 95% in Sub-Saharan Africa (Thresh et al., 1994) that contribute to food insecurity and poverty across the region.

CMD is caused by one or more of 11 DNA viruses collectively designated as cassava mosaic begomoviruses (CMBs) (Crespo-Bellido et al., 2021). In Africa, nine CMB species are associated with CMD, including African cassava mosaic virus (ACMV). Begomoviruses constitute the largest genus in the *Geminiviridae*, a family of DNA viruses that infect many agriculturally important plant species. Like all geminiviruses, begomoviruses have small, circular DNA genomes that are packaged into virions as single-stranded DNA (ssDNA) (Zhang et al., 2001). Begomovirus genomes also exist as double-stranded DNA (dsDNA) that is involved in viral replication and transcription in infected plants (Hanley-Bowdoin et al., 2013).

CMB genomes consist of two DNA components designated as DNA-A and DNA-B that together encode 9-10 proteins (Li et al., 2015; Gong et al., 2021; Wang et al., 2022; Liu et al., 2023). Other studies have indicated that the coding capacity of begomoviruses is greater than the canonical open reading frames (ORFs) (Lin et al., 2017; Gong et al., 2021), and the number of CMB proteins is likely to be higher. DNA-A encodes proteins involved in viral replication, transcription, encapsidation and countering host defenses, while DNA-B encodes proteins involved in viral cell-to-cell and systemic movement and interactions with host defense pathways. CMBs are transmitted by whiteflies (*Bemisia tabaci* Genn.) and through stem cuttings when infected cassava is used for propagation (Legg et al., 2015; Jacobson et al., 2018).

Begomovirus genomes evolve rapidly through mutation (nucleotide substitutions, insertions, and deletions), recombination, and reassortment (Elena et al., 2014; Nigam, 2021). Viral evolution has been associated with emergence of new and more virulent viruses/strains and increased adaptation to new hosts and new environmental conditions (Pita et al., 2001; Duffy and Holmes, 2009; Nigam, 2021). Co-infections of two or more CMBs are frequent and can result in synergism and increased symptom severity (Pita et al., 2001; Duffy and Holmes, 2009; Aimone et al., 2021b). Synergism between ACMV and a recombinant strain, East African cassava mosaic virus–Uganda (EACMV-UG), was associated with the severe CMD pandemic that spread from Uganda to other sub-Saharan countries (Deng et al., 1997; Zhou et al., 1997). To mitigate the pandemic, cassava breeding programs developed CMD resistance cultivars generally carrying the *CMD2* locus (Rabbi et al., 2014; Sheat and Winter, 2023)

Begomoviruses are often associated with satellite DNAs that are packaged into virions and together form complexes that can increase virulence and diseases severity (Briddon et al., 2003; Mansoor et al., 2003; Leke et al., 2015; Kumar et al., 2023). Three major types of DNA satellites have been described for begomoviruses – betasatellites (Briddon et al., 2003), alphasatellites (Briddon et al., 2018), and deltasatellites (Fiallo-Olivé et al., 2012; Lozano et al., 2016). Alphasatellites and betasatellites are approximately half the size of begomovirus genome components (1,300-1,400 nt), while deltasatellites are about one fourth the size of begomovirus genome components (540-750 nt). Alphasatellites and betasatellites contain single open reading frames, but deltasatellites do not have reading frames despite their relationship to betasatellites (Hassan et al., 2016; Rosario et al., 2016; Briddon et al., 2018). Betasatellites and some alphasatellites counter host defenses by interfering with host gene silencing pathways, leading to increased symptom severity during viral infection (Nawaz-ul-Rehman et al., 2010; Abbas et al., 2019; Yang et al., 2019; Zhao et al., 2022).

Two novel DNAs, designated SEGS-1 (sequences enhancing geminivirus symptoms; DNA-II; GenBank accession no. AY836366) and SEGS-2 (DNA-III; AY836367; Sequences Enhancing Geminivirus Symptoms), were isolated from cassava plants showing severe, atypical CMD symptoms in fields near the Tanzanian coast (Ndunguru et al., 2016). Both SEGS contain by GC-rich regions but only share 23% overall sequence identity with each other. Studies in cassava and Arabidopsis established that SEGS-2 is a novel begomovirus satellite that increases disease severity (Ndunguru et al., 2016; Aimone et al., 2021a). Similar to betasatellites, SEGS-2 is a circular DNA of about 1200 nt in size that replicates in infected plant cells in the presence of a helper begomovirus and is packaged into virions in infected plants and whiteflies. SEGS-2 also encodes a single open reading frame that is necessary for disease enhancement. However, the DNA sequence of SEGS-2 and its open reading frame show no relationship to betasatellites and only has homology to a 28-bp sequence in the replication origins of alphasatellites. In contrast, sequences in the cassava genome show 89% identity to SEGS-2 over most of its length, suggesting that the SEGS-2 satellite may have resulted from a recombination event between sequences in an alphasatellite and the cassava genome.

SEGS-1 also occurs as low copy number episomes in CMD-infected cassava and enhances disease symptoms. However, unlike SEGS-2, it can overcome endogenous CMD2 resistance. SEGS-1 shows no sequence relationship with begomovirus satellites or their helper viruses. Instead, SEGS-1 is related to sequences in the cassava genome, which contains a full-length 1007-bp copy of SEGS-1 (99% identify) as well as many other SEGS-1 related sequences (Ndunguru et al., 2016). SEGS-1 genomic sequences have been found in all cassava cultivars examined to date. SEGS-1 episomes have only been found in plants and not in virions or whiteflies, suggesting they may originate from the cassava genome. Given the widespread and potentially universal occurrence of SEGS-1 sequences in the cassava genome, it is important to ask if SEGS-1 activity depends on the formation of an episome or if it can function in a plant genomic context. In the experiments reported here, this question was addressed by using *Arabidopsis thaliana* infected with a CMB as a model system.

## Materials and methods

### SEGS-1 clones

**Figure 1:**
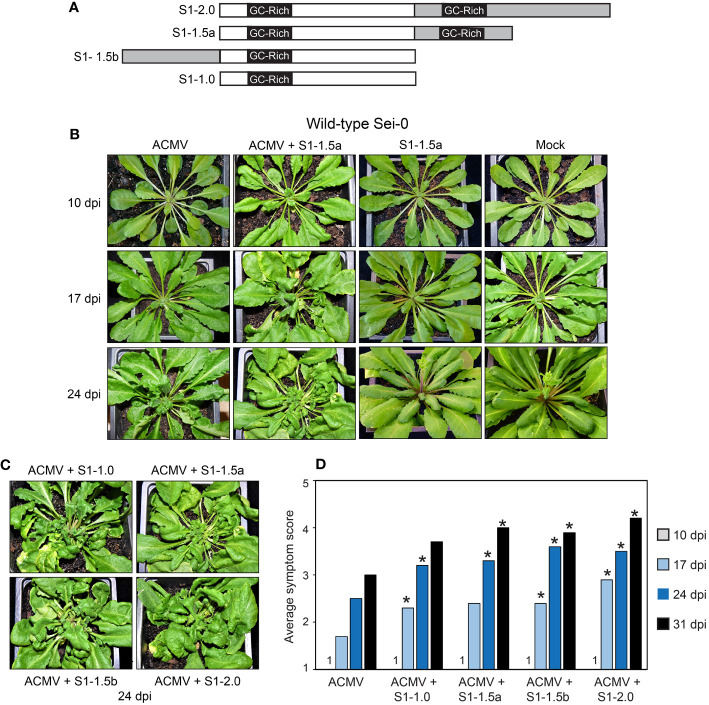
SEGS-1 enhances ACMV symptoms in Arabidopsis Sei-0. **(A)** SEGS-1 clones used for infection studies. The clones include a dimer (S1-2.0), a 1.5-mer with 2 GC-rich regions (S1-1.5a), a 1.5-mer with 1 GC-rich region (S1-1.5b), and a monomer (S1-1.0) that is configured like the full-length copy of SEGS-1 in the cassava genome. The gray segments and an embedded GC-rich region represent sequences that are duplicated in a construct. **(B)** Symptom development in plants inoculated with ACMV alone, ACMV + S1-1.5a, and S1-1.5a alone, or mock (ACMV DNA-B only). **(C)** Symptoms at 24 dpi in plants co-inoculated with ACMV and S1-1.0, S1-1.5a, S1-1.5b, or S1-2.0. **(D)** Time course of average symptom scores (1- no symptoms, 5- severe symptoms) for plants inoculated with ACMV alone or co-inoculated with ACMV + SEGS-1 clone. Values represent the mean of 10 plants per treatment. Asterisks (*) indicate significant differences between ACMV alone and ACMV+SEGS-1 treatments (p < 0.05 in a Wilcoxon ranked sum test).

SEGS-1 clones used in these studies (**Figure 1A**) were generated from a pGEM-T Easy plasmid harboring a dimeric copy of SEGS-1 (pGEM-SEGS-1) described in Ndunguru et al. (2016). The dimeric construct was digested with *Eco*RI and cloned into pUC119 to make pNSB2136 (referred to here as S1-2.0, a dimer of SEGS-1 in pUC119). pGEM-SEGS-1 was also digested with *Pst*I/*Bcl*I to isolate two 500-bp fragments, which were cloned individually into pUC119 previously digested with *Bam*HI/*Pst*I. The resulting 0.5-mer clones were designated as pNSB1827 with the GC-rich sequence and pNSB1828 without the GC-rich sequence from SEGS-1. pNSB2136 was digested with *Pst*I to release a SEGS-1 monomer fragment. To make S1-1.5a (pNSB1829, a partial tandem copy of SEGS-1 with 2 copies of the G-rich region), pNSB1827 was linearized with *Pst*I and ligated with the SEGS-1 monomer fragment. To make S1-1.5b (pNSB1830, a partial tandem copy of SEGS-1 with one copy of the G-rich region), pNSB1828 was digested with *Pst*I and ligated to the SEGS-1 monomer fragment. To make S1-1.0 (pNSB2003), a SEGS-1 monomer fragment was released from pNSB1830 by *Kpn*I digestion, end repaired using Klenow (DNA polymerase I), and then cloned into pUC119. S1-1.0 has a single copy of SEGS-1 that does not include primer sequences introduced during construction of the original pGEM-SEGS-1 clone. All clones were confirmed by Sanger sequencing

*Not*I sites flanking the SEGS-1 monomer insert in pNSB2003 were created sequentially with the QuikChange II Site-Directed Mutagenesis Kit (Agilent Technologies, United States) and the primer pairs, S1for*Not*1-1/S1for*Not*1-2 and S1rev*Not*I-1/S1rev*Not*1-2 (**Table 1**). The mutagenized DNA was transformed into *Escherichia coli* DH5α. Plasmid DNA was extracted from the transformant and digested with *Not*I to release a 1-kb SEGS-1 monomer fragment flanked by *Not*I sites. The resulting fragment was ligated into pMON721 previously linearized with *Not*1 and treated with calf alkaline phosphatase, and transformed into *E. coli* DH5α. DNA from transformants was screened by PCR using the primer pairs, S1.3F/pMON721-R and pMON721-F2/S1.3F (**Table 1**) to establish its orientation in the T-DNA plasmid. Plasmids in the forward (T-S1-1.0F, pNSB2000F) and reverse (T-S1-1.0R; pNSB2000R) orientations were transformed into *Agrobacterium tumefaciens* ABI (Shaw, 1995).

**Table 1:** Primers used in this study.

(A) ACMV DNA-A Primers				
Primer name	Sequence (5’-3’)	Annealing (°C)	Product size (bp)	
ACMV divLF	GACAAGATCCACTCTCCTACGC	58	1397	End point PCR
ACMV divLR	CACATTGCGCACTAGCAACGACTT			
CMAFor4	ATCTGTAAGGTGATTAGTGATGTGA	48	245	End point PCR
CMARev4	ATTGTTGCAGTACTGGGCTCATTATC			
P3P-AA2F	TCTGCAATCCAGGACCTACC	53	165	qPCR
P3P-AA2R+4R	GGCTCGCTTCTTGAATTGTC			
ACMV 400F	CTCAGATGTCAAGTCCTATC	58	415	*In situ* hybridization probe
ACMV 400R	ATTGTGTGGGCCTAAAG			
(B) SEGS-1 primers				
Primer name	Sequence (5’-3’)	Annealing (°C)	Product size (bp)	
1-4F	GGGTAGCCTCTAATCCTTCA	55	587	Episome detection
1-2R	CAGTTGAACTGCTGAACTGC			
(C) Cloning primers				
Primer name	Sequence (5’-3’)	Annealing (°C)	Product size (bp)	
S1for*Not*1-1	CGGCCAGTGAATTCGAGCGGCCGCGCTGGGTACCACTACGC	53		Mutagenesis for transgene construction
S1for*Not*1-2	GCGTAGTGGTACCGAGCGCGGCCGCTCGAATTCACTGGCCG			
S1revNotI-1	ACGACGGCCAGTGAATTCGAGGCGGCCGCCTCGGTACCACTACGCTACGC			
S1.3F	AGGACCTTTGGAGCTCGA	52	595	Colony PCR for transgene orientation
pMON721-R	CATAAGTGCGGAGACGATAGT			
pMON721-F2	CTCATCTGTCAGTGAGGGCCAAG	52	750	
S1.3F	AGGACCTTTGGAGCTCGA			

**(A)** Primer pairs used to detect, quantify, and localize ACMV DNA-A in infected plants (Aimone et al., 2021a). **(B)** Divergent primer pair that amplifies across the SEGS-1 episomal junction and does not amplify the transgene (Ndunguru et al., 2016). **(C)** Primers used to add Not-1 sites to SEGS-1 sequences prior to cloning and to orient SEGS-1 transgene in T-DNA of transformation vector.

### Plant inoculation, sample collection and DNA isolation

For infection studies, Arabidopsis Sei-0 plants were grown at 20°C, 80% humidity and an 8:16 light/dark cycle for 3-4 weeks (about 12 leaves per plant) before inoculation. Plants were then inoculated at 30 psi with a microdrop sprayer (Venganza, Inc.) to deliver gold particles coated with plasmid DNA corresponding to viral infectious clones and SEGS-1 constructs as described earlier (Aimone et al., 2021a). Plants were inoculated with 1.5 µg of cloned SEGS-1 DNA (S1-1.0, S1-1.5a, S1-1.5b and S1-2.0) and with 0.75 µg of each viral replicon plasmid corresponding to ACMV DNA-A or DNA-B (Accession Numbers: MT858793.1 and MT858794.1) (Hoyer et al., 2020).

Wild-type Sei-0 plants were inoculated in six treatment groups; mock (DNA-B alone), ACMV (DNA-A and DNA-B), ACMV + S1-1.0, ACMV + S1-1.5a, ACMV + S1-1.5b and ACMV + S1-2.0. For the transgenic Sei-0 experiments, 3 genotypes were used: i) wild-type Sei-0; ii) transgenic Sei-0 carrying a forward SEGS-1 transgene (T-S1-1.0F); and iii) transgenic Sei-0 carrying a reverse transgene (T-S1-1.0R). Each genotype had two treatment groups – mock (ACMV DNA-B alone) and infected (ACMV DNA-A and DNA-B). For both types of experiments, each treatment consisted of 10 plants, and the experiment was repeated 3 times.

Plants were inspected at 10, 17, 24 and 31 dpi for symptom appearance, which were recorded using a symptom severity scale ranging from 1 (no symptoms) to 5 (severe symptoms). Samples for viral DNA analysis were collected from apical leaf 2 (about 1 cm in length) relative to the rosette center, frozen in liquid nitrogen, and stored at −80°C until DNA extraction. Total DNA was isolated using the CTAB protocol (Doyle and Doyle, 1990) and treated with RNase A (0.1 μg/μL) according to the manufacturer instructions (Thermo Scientific™). DNA concentrations were quantified using a NanoDrop ND-2000 (NanoDrop Technologies).

### Viral DNA and SEGS-1 episome analysis

Viral DNA accumulation was monitored in co-inoculated plants at 24 dpi by end-point PCR using the ACMV DNA-A primer pairs, ACMV divLF/ACMV divLR or CMAFor4/CMARev4 (**Table 1**). A 50-µL PCR reaction contained 100 ng of total DNA, 0.05 U of Standard Taq Polymerase (NEB), 0.2 µM of each primer, and 1× PCR buffer. PCR conditions were initial denaturation at 95°C for 5 min followed by 30 cycles of denaturation for 30 s at 94°C, annealing for 45 s and extension for 1 min. The annealing and extension temperatures were 58/72°C for the ACMV divLF/ACMV divLR primer pair and 48/68°C for the CMAFor4/CMARev4 primer pair. The PCR products corresponding to ACMV DNA-A were resolved by agarose gel electrophoresis. The copy number of ACMV DNA-A (primer pair - P3P-AA2F and P3P-AA2R+4R; **Table 1**) in wild-type and SEGS-1 transgenic plants was determined at 10, 17, and 24 dpi by quantitative PCR using a standard curve as described previously (Aimone et al., 2022).

To assess the presence of SEGS-1 episomes in Arabidopsis plants, total DNA (50 ng) was used as template for rolling cycle amplification (RCA) with EquiPhi29 polymerase Kit (ThermoFisher Scientific, USA) as described by (Aimone et al., 2021b). The RCA product was diluted 10-fold with DNase-free water, and 1 μL was used in a 50-μL PCR reaction containing the divergent primer pair, 1-4F and 1-2R (**Table 1**), using previously established conditions (Ndunguru et al., 2016).

### Viral DNA replication assays

Protoplasts were prepared from *Nicotiana tabacum* NT-1 cells, electroporated with ACMV DNA-A in presence of the designated SEGS-1 clone or pUC119 (negative control), and cultured as described previously (Fontes et al., 1994). The transfections included 1.5 μg of ACMV DNA-A and 10 μg of a SEGS-1 plasmid DNA in the following treatments: mock (no virus), ACMV DNA-A + pUC119, ACMV DNA-A + S1-1.0, ACMV DNA-A + S1-1.5a, ACMV DNA-A + S1-1.5b and ACMV DNA-A + S1-2.0. Total DNA was purified 48 h post transfection, and 30 μg was digested with *Dpn*I and linearized with *Bsu*36I. Viral DNA was resolved using native gel conditions followed by DNA blotting using a ^32^P-labeled ACMV DNA-A probe (948-bp *Nco*I/*Bam*H1 fragment from MT858793.1) (Hoyer et al., 2020). Blots were scanned by using a PhosphorImager and quantified by using IQMacV1.2 software (Storm; Amersham, Inc.). DNA gel blot analysis resolved nascent viral DNA from input plasmid DNA based on size. We were unable to quantify nascent viral DNA by qPCR due to background caused residual input ACMV-A DNA plasmid still present after exhaustive *Dpn*I digestion. Residual plasmid DNA also interfered with assessment of SEGS-1 episomes in protoplasts.

### *In situ* hybridization

Leaf 4 relative to the center of the Arabidopsis rosette was harvested, fixed using paraformaldehyde, and embedded into low-melting-point agarose gel in phosphate buffered saline (PBS) as described by (Shen and Hanley-Bowdoin, 2006). The leaf was cut into 100-μm sections using a Leica VT1000S vibratome (Leica Microsystems). *In situ* hybridization was performed as described by (Aimone et al., 2021a), using a digoxigenin-labeled probe corresponding to 415 bp of the ACMV AC1 gene that was generated using the primer pair ACMV 400F and ACMV 400R (**Table 1**). Virus-positive nuclei were counted in 4 replicate images (2 each from 2 independent experiments) for each treatment, and the treatments were compared using two-tailed paired Student’s t-tests.

## Results

### SEGS-1 enhances ACMV symptoms in Arabidopsis

The cassava genome contains a full-length copy of SEGS-1 with 99% identity to the cloned SEGS-1 sequence and 17 partial sequences with >70% identity (confirmed for cassava reference genome v8.1, (Ndunguru et al., 2016)), making it difficult to determine how SEGS-1 enhances begomovirus disease and overcomes resistance in cassava (Ndunguru et al., 2016). To address this constraint, we asked if SEGS-1 impacts ACMV infection in Arabidopsis, which does not have SEGS-1 related sequences in its genome. For these studies, we used the hypersusceptible Arabidopsis accession, Sei-0 (Lee et al., 1994), because it can be infected by ACMV and shows a similar response to SEGS-2 as cassava (Aimone et al., 2021a). ACMV is not well adapted to Arabidopsis, resulting in variation in the timing of symptoms between experiments. Because of this variation, we assayed 10 plants/treatment and only compared treatments within an experiment. Our conclusions were based on three independent experiments that showed the same relative trends between treatments.

To assess the effect of SEGS-1 on ACMV infection in Arabidopsis, we made a series of clones with different configurations of the SEGS-1 sequence in a pUC119 background (**Figure 1A**). They were generated from the cloned SEGS-1 sequence originally amplified from CMB-infected cassava using a betasatellite universal primer (Ndunguru et al., 2016). The S1-2.0 construct is a tandem dimer of SEGS-1, while S1-1.0 has a single copy. The partial tandem dimers, S1-1.5a and S1-1.5b, have different halves of SEGS-1 duplicated.

**Figure 2:**
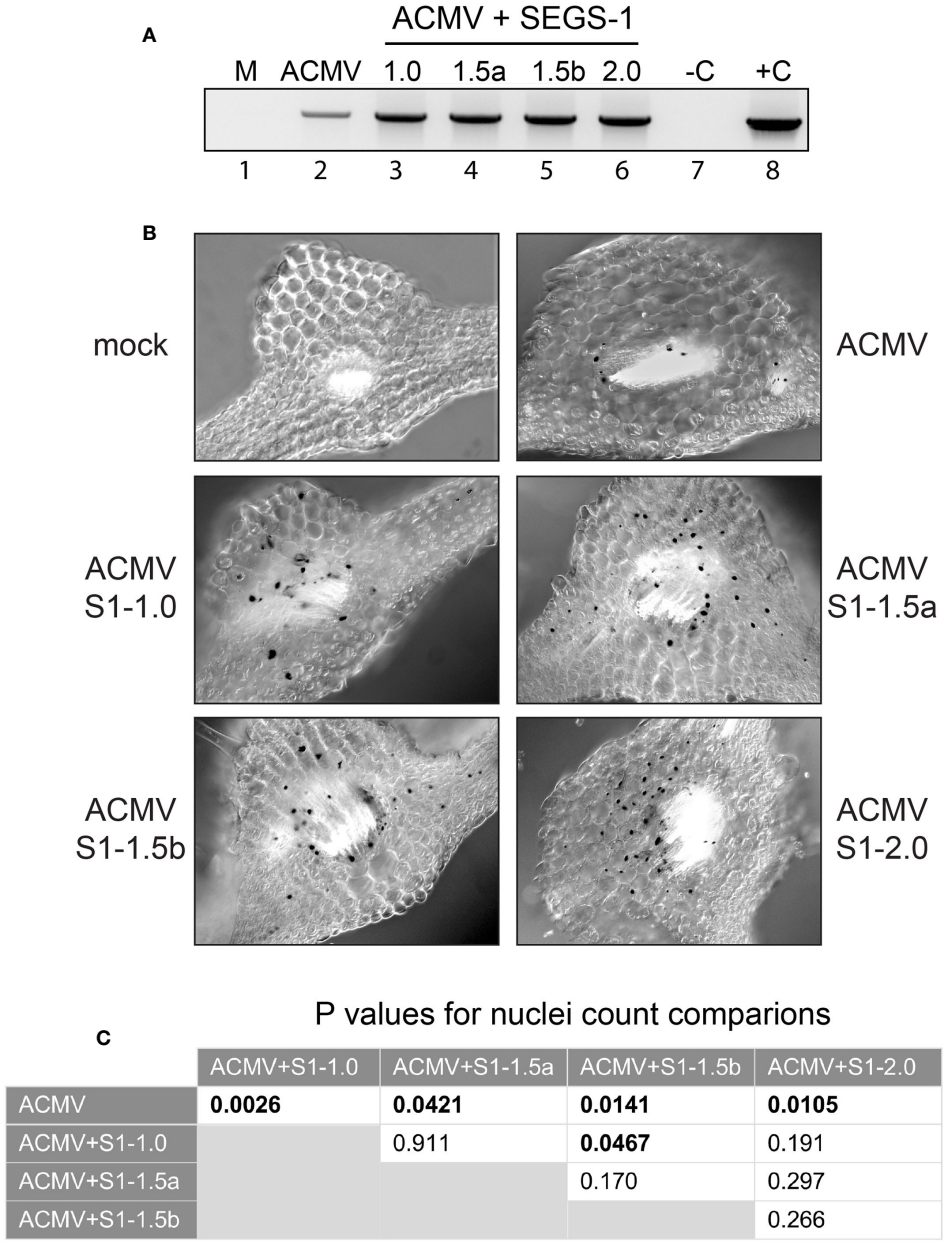
SEGS-1 increases ACMV DNA accumulation in Arabidopsis Sei-0. **(A)** End-point PCR using the ACMV divLF/ACMV divLR primer pair to amplify ACMV DNA-A in mock (M; lane 1), ACMV alone (lane 2), or ACMV co-inoculated with S1-1.0 (lane 3), S1-1.5a (lane 4), S1-1.5b (lane 5) or S1-2.0 (lane 6) at 24 dpi. A negative no template control and a cloned positive plasmid DNA control are indicated by –C and +C (lanes 7 and 8, respectively). **(B)** *In situ* hybridization of ACMV DNA-A in plants at 24 dpi with ACMV alone or co-inoculated with ACMV and the indicated SEGS-1 clone. The *415*-bp, DIG-labeled, DNA-A-specific probe forms a black precipitate over virus-positive nuclei. The leaf sections correspond to regions with vascular bundles where ACMV localizes. Mock plants were inoculated with ACMV DNA-B alone and did not contain infected cells. **(C)** Statistical analyses of virus-positive nuclei counts (**Supplementary Table 1**) from *in situ* hybridization images using two-tailed paired Student’s t-test. Values in bold indicate significant differences (P<0.05).

Sei-0 plants were inoculated with infectious clones corresponding to ACMV DNA-A and DNA-B with or without plasmids with the different SEGS-1 constructs (**Figure 1A**). All the plasmids for an inoculation were co-precipitated onto gold beads to ensure co-delivery. Arabidopsis plants infected with ACMV developed symptoms that included leaf curling, deformation, and stunting (**Figures 1B, C**). Symptoms were not observed at 10 dpi but were apparent at 17, 24, and 31 dpi for all treatments. Plants co-inoculated with ACMV + SEGS-1 displayed more severe symptoms than plants inoculated with ACMV alone. This effect was seen for all four SEGS-1 constructs, indicating that configuration of the SEGS-1 constructs did not affect the outcome. This conclusion is supported by symptom severity score data using a scale from 1 (no symptoms) to 5 (severe symptoms). At 17, 24 and 31 dpi, plants inoculated with ACMV + SEGS-1 had significantly higher symptom scores compared to plants inoculated with ACMV alone (p < 0.05 in a Wilcoxon ranked sum test) (**Figure 1D**). The only exceptions were S1-1.5a at 17 dpi and S1-1.0 at 31 dpi, both of which showed higher symptom scores than ACMV alone but had larger sample-to-sample variation. End-point PCR analysis of total DNA samples collected at 24 dpi gave consistently stronger signals for viral DNA from plants co-inoculated with ACMV and a SEGS-1 construct than ACMV alone (**Figure 2A**, cf. lanes 3-6 to lane 2).

We then used *in situ* hybridization to ask if the enhanced symptoms and higher ACMV DNA accumulation in the presence of SEGS-1 reflected changes in the infection pattern. Sections from infected leaves were subjected to hybridization using a digoxigenin-labeled ACMV DNA-A probe, which selectively binds to viral DNA, along with an anti-digoxigenin detection system that results in the staining of virus-positive nuclei with a black precipitate. More virus-positive cells were observed in the vascular bundles of plants co-inoculated with ACMV and SEGS-1 compared to ACMV alone (**Figure 2B**). This conclusion was supported by nuclei count data (**Supplementary Table 1**) showing that the numbers of virus-positive nuclei were significantly higher when plants were co-inoculated with ACMV and a SEGS-1 construct when compared to the ACMV alone (p < 0.05; **Figure 2C**). This observation is consistent with ACMV infecting more cells or accumulating to detectable levels in more cells in the presence of SEGS-1. No virus was observed outside of the vascular parenchyma indicating that ACMV is limited to vascular bundles in Arabidopsis with or without SEGS-1. No staining was observed in sections from the mock inoculated controls, demonstrating the specificity of the *in situ* assay.

### SEGS-1 increases viral DNA accumulation in tobacco suspension cells

**Figure 3:**
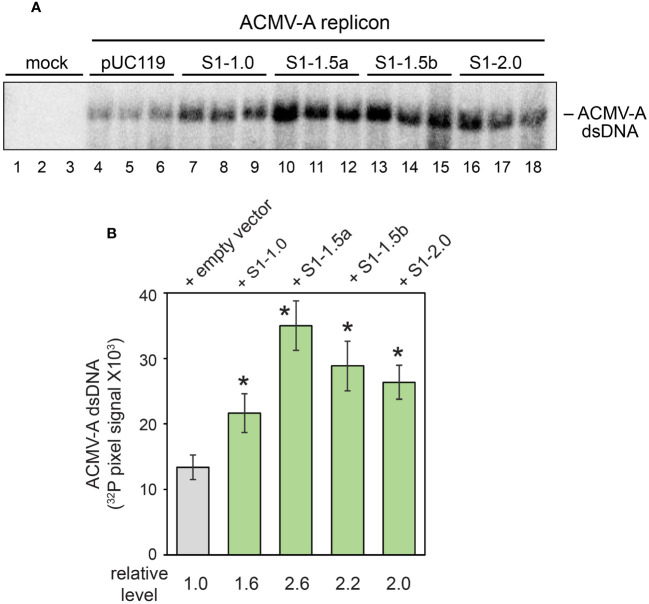
SEGS-1 enhances ACMV DNA-A accumulation in tobacco protoplasts. **(A)** DNA gel blot showing the accumulation of nascent double-stranded ACMV DNA-A in protoplasts from NT-1 suspension cells at 48 h post transfection. The transfections are mock (pUC119: empty vector control), DNA-A + pUC119 (lanes 4-6), DNA-A + S1-1.0 (lanes 7-9), DNA-A + S1-1.5a (lanes 10-12), DNA-A + S1-1.5a (lanes 13-15) and DNA-A + S1-2.0 (lanes 16-18). The blot was hybridized to a 947-bp ACMV DNA-A fragment labeled with ^32^P and visualized by phosphor imaging. **(B)** ^32^P pixels were quantified using GelQuant software. Values represent the mean of 3 replicates/treatment. Bars correspond to ± 2 standard errors from the mean. Asterisks (*) indicate significant differences between the ACMV + empty vector treatment and an ACMV + SEGS-1 treatment (p < 0.05 in a two-tailed Student’s t test).

We asked if SEGS-1 affects viral DNA accumulation in tobacco suspension cells (NT-1) that support viral replication (Fontes et al., 1994). Protoplasts prepared from the NT-1 cell culture were co-transfected with an ACMV DNA-A and pUC119 (empty vector) or plasmids carrying SEGS-1 sequences. Viral DNA accumulation was monitored at 48 h post transfection on DNA gel blots hybridized to a ^32^P-labeled ACMV-A probe. Each treatment was performed in triplicate within an experiment, and the experiment was replicated three times. Higher levels of double-stranded ACMV-A DNA were detected in the treatments containing the SEGS-1 plasmids (**Figure 3A**). The increases in viral DNA were significant (p < 0.05 in a two-tailed Student’s T-test) in comparisons between each SEGS-1 plasmid to the pUC119 control (**Figure 3B**). No single-stranded ACMV-A DNA was detected on the gel blots, most likely because NT-1 cells do not support repression of the AC1 promoter, a prerequisite for coat protein production and sequestration of single-stranded viral DNA (Hanley-Bowdoin et al., 2013; Rizvi et al., 2015; Wu et al., 2021).

### A SEGS-1 transgene enhances ACMV infection in Arabidopsis

**Figure 4:**
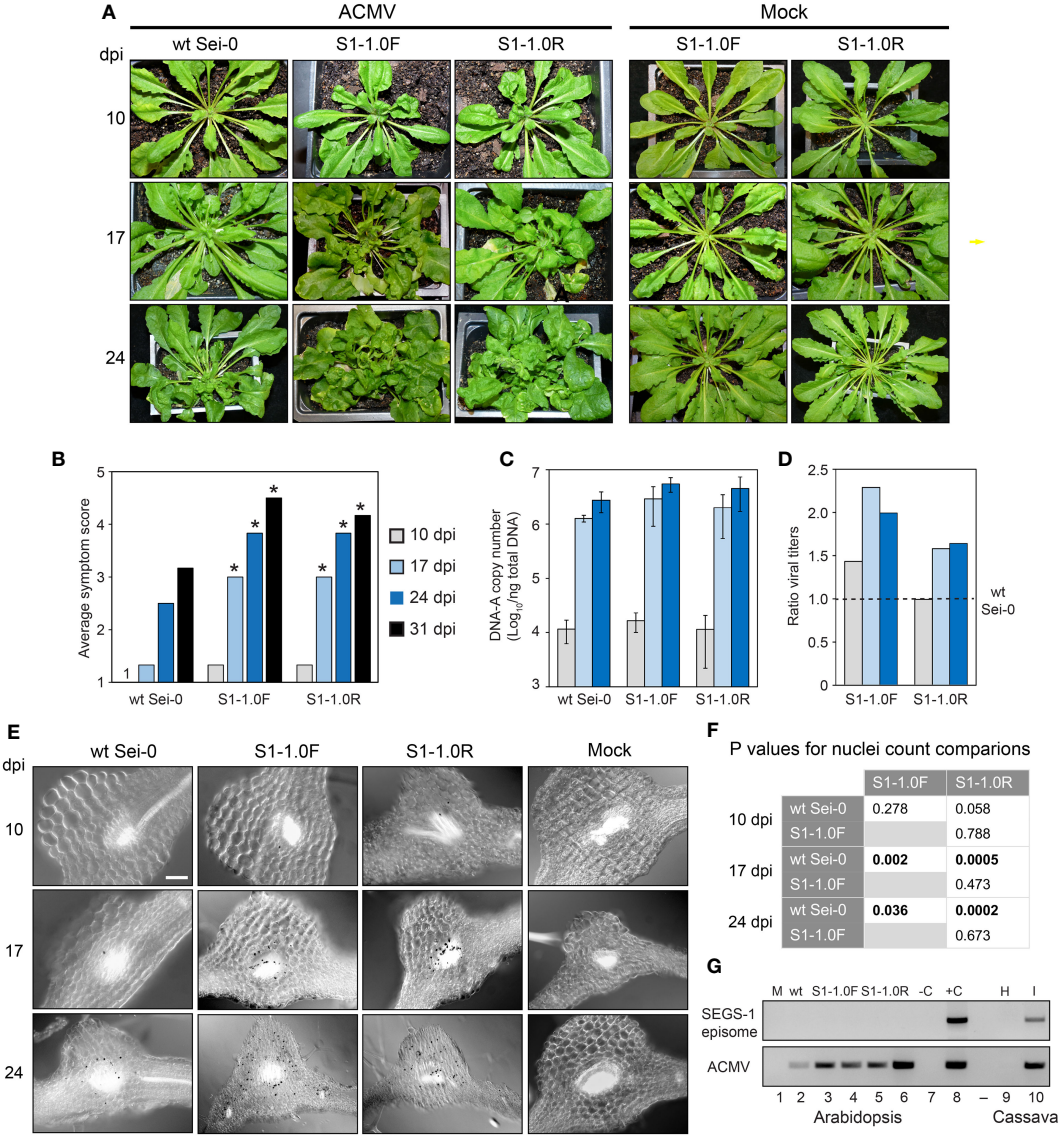
A SEGS-1 transgene enhances ACMV infection in Arabidopsis Sei-0 plants. **(A)** Time course (10, 17 and 24 dpi) of symptom development after inoculation with ACMV DNA-A + DNA-B or ACMV B alone (mock) in wild-type plants and in transgenic plants carrying a monomeric SEGS-1 transgene in the forward (S1-1.0F) or a reverse (S1-1.0R) orientation. **(B)** Time course of average symptom scores for wild-type Sei-0, S1-1.0F and S1-1.0R plants inoculated with ACMV at 10, 17, 24 and 31 dpi. Values represent the mean of 10 plants per treatment. Asterisks (*) indicate significant differences between ACMV alone and ACMV+SEGS-1 treatments (p < 0.05 in a Wilcoxon ranked sum test). **(C)** ACMV DNA-A copy number/ng total DNA in infected wild-type, S1-1.0F and S1-1.0R plants. The values represent the mean of 4 plants/treatment. Bars correspond to ± 2 standard errors from the mean. The ACMV DNA-A copy numbers in S1-1.0F and S1-1.0R plants were higher than in wild-type plants at 17 and 24 dpi, but no significant differences between the means were detected between the treatments by two-tailed Student’s t tests. The bars represent ± 2 standard errors from the mean. **(D)** Ratios of ACMV DNA-A mean copy numbers in S1-1.0F or S1-1.0R plants relative to wild-type plants. The dotted line represents the copy number in wild-type plants set to 1. **(E)** *In situ* hybridization of ACMV DNA-A in wild-type Sei-0, S1-1.0F and S1-1.0R plants at 10, 17 or 24 dpi with ACMV. The 415-bp DIG-labeled, DNA-A-specific probe forms a black precipitate over virus-positive nuclei. The leaf sections correspond to regions with vascular bundles where ACMV localizes. Mock plants were inoculated with ACMV DNA-B and did not contain infected cells. **(F)** Statistical analyses of virus-positive nuclei counts (**Supplementary Table 2**) from *in situ* hybridization images using two-tailed paired Student’s t-test. Values in bold indicate significant differences (P<0.05). **(G)** SEGS-1 episome analysis. The divergent primer pair 1-4F/1-2R (**Table 1**) was used to detect SEGS-1 episomes after RCA of total DNA. The top gel shows no PCR products amplifying across the SEGS-1 episome junction in ACMV-inoculated wild-type (wt; lane 2), S1-1.0F (lanes 3 and 4), and S1-1.0R (lanes 5 and 6) plants. C- is the water only negative PCR control (lane 7). C+ is the positive PCR control using SEGS-1 plasmid DNA as template that amplified in parallel with the Arabidopsis samples (lane 8). The bottom gel shows end-point PCR analysis using the CMAFor4/CMARev4 primer pair to amplify ACMV DNA-A in the same Arabidopsis DNA samples. Mock (lane 1) is DNA from an Arabidopsis plant inoculated with ACMV DNA-B only. In lanes 9 and 10, DNA samples from uninfected and ACMV-infected cassava plants were analyzed in parallel using the same protocol as the Arabidopsis episome assays. SEGS-1 episomes were detected in the DNA sample analyzed in lane 10 but not in the sample in lane 9.

We asked if SEGS-1 enhances ACMV infection when it is integrated into a plant genome, as it is in the cassava genome. For these experiments, we generated transgenic Arabidopsis Sei-0 lines carrying a single copy of SEGS-1, analogous to the full copy of SEGS-1 in the cassava genome. Homozygous T_3_ plants carrying the forward or reverse T-DNA orientation of monomeric SEGS-1 (S1-1.0F and S1-1.0R, respectively, **Table 2**) appeared phenotypically normal (**Figure 4A**, mock), indicating that the SEGS-1 sequences by themselves do not impact Arabidopsis. These observations are consistent with the previous result that inoculation of SEGS-1 DNA by itself has no effect on Arabidopsis plants (**Figures 1B**, **S1-1.5A**).

**Table 2:** Clones used in this study.

(A) Infectious clones				
Name	Insert	Cloning vector	Clone name	Description
pILTAB409	ACMV DNA-A	pBluescriptIIKS-	pILTAB409	Partial tandem dimer of ACMV DNA-A
pILTAB411	ACMV DNA- B	pBluescriptIIKS-	pILTAB411	Partial tandem dimer of ACMV DNA-B
(B) SEGS-1 plasmids				
Name	Insert	Cloning vector	Clone name	Description
S1-1.0	SEGS-1 monomer	pUC119	pNSB2000	Monomer of SEGS-1
S1-1.5a	SEGS-1 1.5a	pUC119	pNSB1829	Partial tandem dimer of SEGS-1 with two GC-rich regions
S1-1.5b	SEGS-1 1.5b	pUC119	pNSB1830	Partial tandem dimer of SEGS-1 with one GC-rich region
S1-2.0	SEGS-1 dimer	pUC119	pNSB2136	Dimer of SEGS-1
(C) Plant transformation plasmids				
Name	Insert	Cloning vector	Clone name	Description
S1-1.0F	SEGS-1 monomer	pMON721	pNSB2000F	SEGS-1 monomer in forward orientation
S1-1.0R	SEGS-1 monomer	pMON721	pNSB2000R	SEGS-1 monomer in reverse orientation

**(A)** ACMV infectious clones that were co-inoculated to initiate infection (Aimone et al., 2021a). **(B)** Clones with different configurations of the SEGS-1 sequence that were co-inoculated with ACMV. **(C)** Clones with the SEGS-1 sequence in opposite orientations in the T-DNA.

We compared ACMV infection in wild-type Sei-0, S1-1.0F, and S1-1.0R plants. The SEGS-1 transgenic plants showed earlier onset of viral symptoms (as early as 10 dpi) and faster disease progression compared to wild-type plants (**Figure 4A**). At 17, 24 and 31 dpi, plants with a SEGS-1 transgene had significantly higher symptom scores compared to wild-type plants (p < 0.05 in a Wilcoxon ranked sum test) (**Figure 4B**). S1-1.0F and S1-1.0R plants had similar symptom scores over the timeframe of the experiment, indicating the orientation of the SEGS-1 sequence in the T-DNA did not impact symptom enhancement.

We also compared the copy number of ACMV DNA-A in wild-type and SEGS-1 transgenic plants by qPCR. At 10 dpi, 10,000 -20,000 copies/ng total DNA were detected in the three genotypes, indicating that viral DNA accumulated before symptom appearance (**Figure 4C**). By 17 dpi, DNA-A copy number was > 1 million/ng total DNA in all three genotypes. The DNA-A copy numbers in transgenic SEGS-1 plants was 1.4 to 2.3-fold greater than in wild-type plants at 17 and 24 dpi (**Figure 4D**). *In situ* hybridization detected more ACMV-positive cells in the SEGS-1 transgenic plants than wild-type Sei-0 (**Figure 4E**). A few infected cells were seen in the vascular bundles at 10 dpi. The numbers of infected cells increased in all three genotypes at 17 and 24 dpi but were significantly higher in the SEGS-1 transgenic plants compared to the wild-type Sei-0 (P<0.05; **Figure 4F**; **Supplementary Table 2**).

### The genomic copy of SEGS-1 is active in Arabidopsis

SEGS-1 episomes have been reported in CMB-infected cassava plants that may have derived from the full-length copy of SEGS-1 in the cassava genome (Ndunguru et al., 2016). Hence, we asked if episomes occur during ACMV infection of the transgenic Arabidopsis plants also carrying a full-length copy of SEGS-1 in their genomes. Total DNA was isolated at 24 dpi from ACMV-infected wild-type Sei-0 plants and plants carrying a SEGS-1 monomer transgene (S1-1.0F or S1-1.0R). The DNA was subjected to rolling circle amplification (RCA) followed by PCR using divergent primers that amplify across the junction formed in SEGS-1 episomes as a result of circularization (**Table 1**). The primers do not amplify a linear copy of SEGS-1 integrated into the cassava genome (Ndunguru et al., 2016) or in the Arabidopsis genome. Using the same amplification protocols that successfully detected SEGS-1 episomes in infected cassava (**Figure 4G**, lane 10) and SEGS-2 episomes in Arabidopsis plants (Aimone et al., 2021a), we found no evidence of SEGS-1 episomes in infected Arabidopsis plants carrying the SEGS-1 transgene in either orientation (lanes 2-6). The same plants displayed severe symptoms and were positive for ACMV DNA-A by end-point PCR analysis (**Figure 4G**). We did not observe evidence of SEGS-1 episomes like those formed in cassava in more than 30 Arabidopsis plants from three independent experiments. These results established that SEGS-1 activity is not mediated by an episome in transgenic Arabidopsis. Instead, the SEGS-1 transgene is active in a host chromosomal context in Arabidopsis.

## Discussion

Many begomoviruses associate with episomal DNAs that are not essential for infection but often influence disease processes. These episomes are generally satellites that are transmitted as part of a begomovirus complex (Nawaz-ul-Rehman et al., 2021; Fiallo-Olivé and Navas-Castillo, 2023). The presence of a satellite can increase pathogenicity, overcome plant resistance, and influence virus movement and host range (Gnanasekaran et al., 2019; Nawaz-ul-Rehman et al., 2021; Fiallo-Olivé and Navas-Castillo, 2023). An earlier study showed that SEGS-1 and SEGS-2 episomes occur in CMB-infected cassava, raising the possibility that they are begomovirus satellites (Ndunguru et al., 2016). Recently, we reported that SEGS-2 is a circular, single-stranded DNA molecule that replicates during infection in the presence of ACMV DNA-A and is packaged into virions in infected plants and whiteflies (Aimone et al., 2021a). We also showed that SEGS-2 encodes an open reading frame that is required for enhancement of CMB infection. These properties are consistent with the identification of SEGS-2 as a novel begomovirus satellite that is part of the CMB complex. In contrast, SEGS-1 DNA is not packaged into virions in infected cassava plants and cannot be detected in whiteflies that fed on plants with SEGS-1 episomes (Ndunguru et al., 2016). In this report, we show that SEGS-1 DNA enhances ACMV infection in Arabidopsis Sei-0 when applied exogenously or integrated into the host genome. Moreover, no SEGS-1 episomes were detected in ACMV-infected Arabidopsis plants with a SEGS-1 transgene. These results established that SEGS-1 activity is not mediated by an episome in transgenic Arabidopsis. Instead, the SEGS-1 transgene is active in a chromosomal context in Arabidopsis. All available evidence (Ndunguru et al., 2016) and the results reported here indicate that SEGS-1 is not a satellite.

The ubiquitous nature of SEGS-1 related sequences in the cassava genome has made it difficult to study SEGS-1 in cassava. We addressed this constraint by examining SEGS-1 activity in Arabidopsis, which does not have SEGS-1 related sequence in its genome. Arabidopsis plants co-inoculated with ACMV and exogenous SEGS-1 DNA displayed symptoms sooner and the symptoms became more severe overtime than those observed in plants only inoculated with ACMV. SEGS-1 is also active as a transgene in the Arabidopsis genome, rendering disease enhancement in ACMV-infected plants. SEGS-1 enhances CMB infection in cassava and Arabidopsis similarly but displayed no activity in *Nicotiana benthamiana* (Ndunguru et al., 2016). This difference suggests that the host interactions that mediate SEGS-1 activity are conserved in cassava and Arabidopsis but not in *N. benthamiana*.

The presence of the SEGS-1 transgene in every cell of an Arabidopsis plant resembles the situation in cassava, in which every cell also has a genomic copy of SEGS-1. However, unlike cassava, we found no evidence of SEGS-1 episomes in Arabidopsis during infection even though the configuration of the transgene was the same as the linear full-length copy in the cassava genome. We cannot rule out that generation of SEGS-1 episomes in CMB-infected cassava depends on sequences outside the SEGS-1 full copy that are missing in the transgene. Independent of this possibility, our results clearly showed that SEGS-1 activity does not require episome formation in Arabidopsis and, instead, is mediated directly by the transgene from a genomic context. These observations are striking because unlike the transgenic Arabidopsis plants, SEGS-1 enhancement is not a universal feature of CMB infection in cassava as one might predict given the ubiquitous nature of SEGS-1 sequences in the cassava genome. A possible explanation for this difference is that the cassava genomic copy of SEGS-1 and the Arabidopsis transgene are in different chromatin environments that differentially impact SEGS-1 activity. The cassava genome contains one full-length copy of SEGS-1, 17 partial copies (≥ 200-bp match; E value ≤10), and hundreds of sequences with shorter and/or weaker matches. Thus, in cassava, SEGS-1 may be perceived as a member of a repetitive sequence family that is inactivated by DNA methylation and/or sequestration into heterochromatin (Biscotti et al., 2015; Ramakrishnan et al., 2022). In Arabidopsis, the SEGS-1 transgene is likely to be inserted into more accessible euchromatin (Kim et al., 2007; Shilo et al., 2017), where it is not silenced because it is not recognized as a repetitive sequence.

A universal feature of begomoviruses is that they interfere with transcriptional gene silencing (TGS) by targeting plant DNA methylation pathways that contribute to the host defense response against DNA viruses (Rodriguez-Negrete et al, 2013; Wang et al., 2018; Wang et al., 2020). Begomovirus proteins have been shown to target enzymes in the host methyl cycle or in DNA methylation pathways directly to repress methylation (Wang et al., 2020; Gui et al., 2022). Suppression of TGS alters the methylation status of the host genome as well as viral DNA (Gui et al., 2022; Zhang et al., 2023). Begomovirus infection has been associated with demethylation and activation of transposable elements in the host genome (Gui et al., 2022; Guo et al., 2022) and similarly might activate the genomic copy of SEGS-1 in cassava. The efficiency of SEGS-1 activation may be affected by many factors including cassava genetic variation, CMB virulence, disease pressure, and environmental conditions (Legg et al., 2015; Ferguson et al., 2019; Houngue et al., 2019; Patil and Fauquet, 2021), such that SEGS-1 is activated in some but not all cassava plants during infection.

Our results provide insight into potential mechanisms whereby SEGS-1 could enhance disease processes. Unlike SEGS-2, SEGS-1 lacks any significant open reading frames, and we could not detect SEGS-1 transcripts in transgenic Arabidopsis. We hypothesize that SEGS-1 might function via a small RNA that was not detected in our mRNA analysis (not shown). *In situ* hybridization studies indicated that SEGS-1 increases the number of virus-positive cells in vascular tissue during infection, suggesting that SEGS-1 might facilitate cell-to-cell movement leading to more infected cells. However, the apparent increase in the number of virus-positive cells could simply reflect the ability to detect more cells due to higher levels of viral DNA per cell. This idea is supported by the observation that ACMV-positive cells are confined to vascular tissue even in the presence of SEGS-1. Moreover, the presence of SEGS-1 is associated with increased accumulation of ACMV DNA-A in tobacco protoplasts that support viral DNA replication but not viral movement (Motoyoshi, 2018; Dai et al., 2022). SEGS-1 could impact viral DNA accumulation by modulating the activity or expression of viral and/or host replication factors (Garcia-Ruiz, 2018) or by suppressing methylation of the viral genome, which interferes with viral replication and transcription (Yang et al., 2011; Gui et al., 2022).

SEGS-1 enhances begomovirus disease symptoms and virus accumulation and changes the dynamics of disease progression to cause early onset of symptoms. The fact that SEGS-1 functions from a transgene in the Arabidopsis genome raises the possibility that the SEGS-1 sequence in cassava genome is also active. SEGS-1 represents a major threat to cassava because all known cultivars contain a genomic copy of SEGS-1. Hence, it is essential to determine how SEGS-1 functions and under what conditions the genomic copy might be activated in cassava either directly or by mobilization of an episome that is active. The studies in Arabidopsis represent a key step in understanding the requirement for SEGS-1 activation.

SEGS-1 and SEGS-2 were initially amplified together from cassava plants showing severe symptoms in Tanzanian fields and were named SatII (DNA-II; GenBank accession no. AY836366) and SatIII (DNA-III; AY836367) respectively. This nomenclature was based on an assumption that both DNAs were satellites that enhance CMD severity. They were later renamed SEGS-1 and SEGS-2 to reflect their capacities more accurately as Sequences Enhancing Geminivirus Symptoms without implying that they were also CMB satellites (Ndunguru et al., 2016). Recent studies have established that SEGS-2 is a novel satellite (Aimone et al., 2021a) and that SEGS-1 can function as a host genomic sequence. Thus, even though their names are nearly identical, it will be important to consider them differently when devising strategies to reduce the effects of SEGS-1 and SEGS-2 on CMD.

## Data availability statement

The original contributions presented in the study are included in the article/**Supplementary Material**. Further inquiries can be directed to the corresponding authors.

## Author contributions

CR and MD performed the experiments and wrote the manuscript. EC and LD performed some of the experiments. EA and FT provided academic mentoring of the research. JN and JA-I conceptualize the studies and provided important input. LHB also conceptualized the studies and helped to assess the results and write the manuscript. All authors contributed to the article and approved the submitted version.
