# Supplementary material for: SEGS-1 a cassava genomic sequence increases the severity of African cassava mosaic virus infection in Arabidopsis thaliana
Source: Front Plant Sci. 2023 Oct 17;14:1250105. doi: 10.3389/fpls.2023.1250105 (PMC10616593; doi:10.3389/fpls.2023.1250105)
Supplement: Supplementary file 1 [file DataSheet_1.pdf]

Supplementary Table 1. Count of virus-positive nuclei in Sei-0 plants co-inoculated with ACMV ± SEGS-1 plasmid DNA

	Mock	ACMV	ACMV+S1-1.0	ACMV+S1-1.5a	ACMV+S1-1.5b	ACMV+S1-2.0
Replicate 1	0	9	22	31	34	40
Replicate 2	0	11	24	19	31	25
Replicate 3	0	7	23	15	48	36
Replicate 4	0	4	24	30	33	23

Supplementary Table 2. Count of virus-positive nuclei in infected wild-type and SEGS-1 transgenic plants

		Mock	wt Sei-0	S1-1.0F	S1-1.0R
10 dpi	Replicate 1	0	0	1	2
	Replicate 2	0	0	0	2
	Replicate 3	0	0	4	2
	Replicate 4	0	0	0	0
17 dpi	Replicate 1	0	1	16	21
	Replicate 2	0	2	20	25
	Replicate 3	0	1	20	21
	Replicate 4	0	1	22	18
24 dpi	Replicate 1	0	13	75	47
	Replicate 2	0	5	41	39
	Replicate 3	0	11	24	43
	Replicate 4	0	7	42	35
